# Maternal steroid levels and the autistic traits of the mother and infant

**DOI:** 10.1186/s13229-021-00453-7

**Published:** 2021-07-08

**Authors:** A. Tsompanidis, E. Aydin, E. Padaigaitė, G. Richards, C. Allison, G. Hackett, T. Austin, R. Holt, S. Baron-Cohen

**Affiliations:** 1 Autism Research Centre, Department of Psychiatry, University of Cambridge, Cambridge, UK; 2 Department of Clinical Psychological Science, Faculty of Psychology and Neuroscience, Maastricht University, Maastricht, The Netherlands; 3 School of Psychology, Faculty of Medical Sciences, Newcastle University, Newcastle upon Tyne, UK; 4 The Rosie Hospital, Cambridge University Hospitals Foundation Trust, Cambridge, UK

**Keywords:** Autism, Autistic Traits, Prenatal, Pregnancy, Estradiol, Sex, Interaction

## Abstract

**Background:**

Prenatal sex steroids have been associated with autism in several clinical and epidemiological studies. It is unclear how this relates to the autistic traits of the mother and how early this can be detected during pregnancy and postnatal development.

**Methods:**

Maternal serum was collected from pregnant women (*n* = 122) before or during their first ultrasound appointment [mean = 12.7 (SD = 0.7) weeks]. Concentrations of the following were measured via immunoassays: testosterone, estradiol, dehydroepiandrosterone sulphate, progesterone; and sex hormone-binding globulin which was used to compute the free fractions of estradiol (FEI) and testosterone (FTI). Standardised human choriogonadotropin (hCG) and pregnancy-associated plasma protein A (PAPP-A) values were obtained from clinical records corresponding to the same serum samples. Mothers completed the Autism Spectrum Quotient (AQ) and for their infants, the Quantitative Checklist for Autism in Toddlers (Q-CHAT) when the infants were between 18 and 20 months old.

**Results:**

FEI was positively associated with maternal autistic traits in univariate (*n* = 108, Pearson’s *r* = 0.22, *p* = 0.019) and multiple regression models (semipartial *r* = 0.19, *p* = 0.048) controlling for maternal age and a diagnosis of PCOS. Maternal estradiol levels significantly interacted with fetal sex in predicting infant Q-CHAT scores, with a positive relationship in males but not females (*n* = 100, interaction term: semipartial *r* = 0.23, *p* = 0.036) after controlling for maternal AQ and other covariates. The opposite was found for standardised hCG values and Q-CHAT scores, with a positive association in females but not in males (*n* = 151, interaction term: *r* = −0.25, *p* = 0.005).

**Limitations:**

Sample size of this cohort was small, with potential ascertainment bias given elective recruitment. Clinical covariates were controlled in multiple regression models, but additional research is needed to confirm the statistically significant findings in larger cohorts.

**Conclusion:**

Maternal steroid factors during pregnancy are associated with autistic traits in mothers and their infants.

**Supplementary Information:**

The online version contains supplementary material available at 10.1186/s13229-021-00453-7.

## Background

Autism is a neurodevelopmental condition characterised by difficulties in social communication alongside unusually restricted interests, repetitive behaviour and frequent sensory hypersensitivity [4]. Co-occurring clinical conditions are common and include learning difficulties, sleep disorders and nutritional intolerances [19]. Diagnosis of autism is possible as early as 18 months of age [27]. Autistic traits exist along a spectrum in the wider population [11]. This can be measured in infancy, as demonstrated by the Quantitative Checklist for Autism in Toddlers (Q-CHAT), a novel, dimensional measure of autistic traits shown to predict later autism diagnosis in several validation studies [2, 3, 43].

Autism is diagnosed more often in males than in females, despite increasing awareness of its differential presentation in females [33]. The reasons for this gender ratio likely reflect both prenatal biology (genetics and sex steroids) and limitations in current diagnostic methods [32]. In the general population, there are on average, sex differences in specific psychological traits (e.g. systemising and empathising), which are shifted towards a “male” profile in autistic individuals of both genders [8, 23].

Several lines of evidence indicate that prenatal sex steroid hormones may be mediating autism likelihood. First, a study of neuroanatomical differences between autistic and neurotypical brains revealed autistic females have atypical structure in regions that substantially overlap with sexually dimorphic regions in neurotypical controls. This suggests that autism affects female brains in regions related to sexual differentiation, which is in turn regulated by prenatal sex steroid hormones [31]. This male-shift is also evident in childhood, as indicated by studies showing that facial features of autistic boys, girls and their siblings are masculinised compared to neurotypical controls [48, 49]. Autistic males also have higher levels of steroid hormones in amniotic fluid, when these were assessed in a multivariate analysis, as well as higher levels of estrogens in particular in univariate analyses of the same amniotic fluid samples [9, 10]. Additionally, estradiol levels are elevated in maternal serum of pregnancies linked to a later autism diagnosis in the child [13].

Traits and developmental profiles related to autism have also been linked to prenatal sex steroids. For example, prenatal levels of testosterone measured during amniocentesis are negatively correlated with the frequency of eye-contact at 12 months [34] and, vocabulary size at 18 and 24 months [35], and are positively correlated with restricted interests, attention to detail [29] and with autistic traits at 18 and 48 months [5, 6].

Epidemiological studies have revealed that maternal polycystic ovary syndrome (PCOS) increases the likelihood of autism in their children [16, 17, 30]. This effect is not primarily driven by shared genetics and is further modulated by the sex of the offspring [15].

PCOS is a complex syndrome affecting between 4 and 20% of women of reproductive age (depending on the diagnostic criteria), impacting their fertility, metabolism and endocrine regulation [44]. It is particularly associated with higher baseline levels of androgens, as well as with a wider endocrine dysregulation during pregnancy (e.g. on placental function) [37]. Steroid-related conditions such as PCOS and placental complications are also more common in autistic people [17, 36, 40], and autistic adults demonstrate signs of steroid imbalance in various tissues [18, 28, 42]. Further research is needed to understand the nature and timing of this endocrine imbalance in autism, as well as to study whether it extends to the wider spectrum of autistic traits in the general population.

The prenatal environment shows baseline sex differences in steroid production via the placenta [21], as well as in markers of placental formation and function, such as human choriogonadotropin (hCG) [1, 20, 38]. Atypical levels in both have been found in maternal serum of pregnancies that resulted in an autistic child, with both higher (for estradiol) and lower levels (for estriol) reported compared to controls [13, 53]. However, these prenatal factors have not been studied together with fetal sex and in relation to both maternal and infant autistic traits.

To evaluate all these prenatal factors, we assessed both the mothers and their infants for autistic traits [via the Autism Spectrum Quotient (AQ) and Q-CHAT, respectively] in a longitudinal cohort. We then tested whether steroid hormone levels (estradiol, testosterone, DHEAS, progesterone) and placental markers (e.g. hCG) in maternal serum collected during the mother’s first ultrasound appointment predicted the autistic traits measured in mothers during pregnancy and in infants at the 18-month follow-up.

## Methods

### Cohort recruitment

Mothers were recruited during their pregnancy, during or immediately before their routine 20-week ultrasound scan [mean gestational age of 20.3 (SD = 0.4) weeks], between 2016 and 2018 at the Rosie Hospital, Cambridge University Hospitals NHS Foundation Trust [7]. This study had been approved by the East of England Cambridge Central Research Ethics Committee (REC Ref 16/EE/0004) and the Research and Development Department of Cambridge University Hospitals. Eligibility inclusion criteria for the study were as follows: (1) little/no consumption of alcohol during pregnancy, (2) no smoking or recreational drug use during pregnancy, and (3) a singleton pregnancy of a fetus (4) whose measurements indicated their size to be appropriate for gestational age [no intrauterine growth restriction (IUGR) or large-for-gestational age (LGA)]. For the postnatal part of the study, the parents of all live singleton births were asked to take part in a series of developmental follow-ups during the first 2 years of life of their infant. Participating mothers also gave informed consent for access to all their pregnancy-related clinical records, test results, and the biological samples that were obtained during their routine clinical care of their pregnancy (both those acquired before as well as after the point of recruitment).

### Clinical data collection

**Fig. 1:**
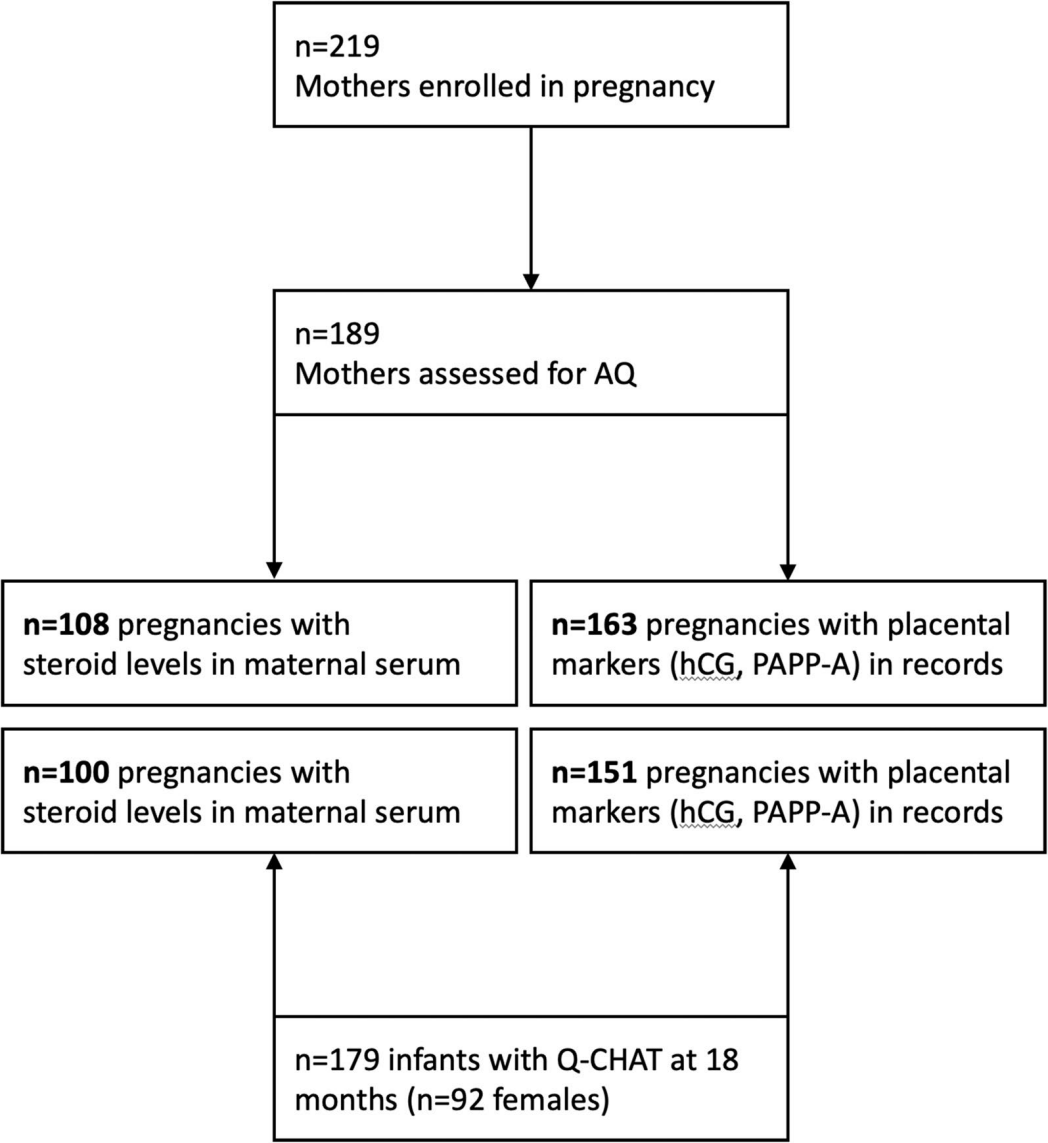
Flow chart of the study, showing different cohort sizes for each comparison of autistic traits (AQ or Q-CHAT) to prenatal measurements (steroid hormones or placental markers)

Serum samples had been collected at the end of the first trimester [mean gestational age of 12.7 (SD = 0.7) weeks since conception] by a specialist phlebotomist at the Rosie Hospital, Cambridge. These were initially assayed for the levels of human choriogonadotropin (hCG) and pregnancy-associated peptide alpha (PAPP-A) as part of a national screening programme for biomarkers of Down’s Syndrome and other conditions, and any remaining serum was stored at – 80 °C. These samples were retrospectively linked to the participating mothers, following their recruitment at approximately 20-weeks gestation. A total of *n* = 122 of these samples were subsequently thawed and transferred to separate vials (1 ml aliquots per sample), which were further anonymised and sent for additional analysis at the Core Biochemical Assays Laboratory (CBAL) at Addenbrookes Hospital, Cambridge. These corresponded to a subset of the study cohort (*n* = 219) (Fig. 1), since, in many instances, serum had been depleted or discarded after routine prenatal testing prior to recruitment.

In addition, mothers were asked to fill in a Pregnancy History Questionnaire (PHQ) at the point of recruitment [mean gestational age of 20.3 (SD = 0.4) weeks]. The PHQ is a self-report inventory designed to collect information on metabolic, reproductive and clinically diagnosed conditions of the mothers pertaining to their current pregnancy, as well as those previous. Maternal hirsutism was ascertained by the question ‘During your adult years, have you found coarse, dark hair, growing in any of the following areas?’, followed by drawings of multiple body areas that are prone to secondary hair growth (e.g. chest, lower face, upper or lower limbs), as described in previous studies [12].

Following labour, birth records containing information on neonate weight and gestational age at birth were also collected.

### Additional assays

The following steroids and peptides were assessed in terms of concentration: Testosterone (T), Estradiol (E2), Dehydroepiandrosterone sulphate (DHEAS), Progesterone (P), sex hormone-binding globulin (SHBG). Samples were analysed on a DiaSorin Liaison® XL automated immunoassay analyser using a one-step competitive chemiluminescence immunoassay for each hormone and two monoclonal antibodies for each peptide. All reagents, standards and consumables are those supplied by DiaSorin [DiaSorin S.p.A, 13040 Saluggia (VC), Italy]. Batch quality precision data and concentration thresholds of detection for each assay are reported in the Additional file 1 (Additional file 1: Table S1).

### Autistic traits

Mothers were asked to complete the Autism Spectrum Quotient (AQ)—Adult version [11] during or immediately after their 20-week routine ultrasound scan at the Rosie Hospital [mean gestational age of 20.3 (SD = 0.4) weeks]. In addition, parents were invited via email to complete an online version of the Quantitative Checklist for Autism in Toddlers (Q-CHAT) [3] after their infant reached 18 months of age (mean = 570.6 days, SD = 21.8 days).

All study data were collected and managed using REDCap electronic data capture tools hosted at the University of Cambridge [24, 25].

### Statistical analysis

Autistic trait distributions (maternal AQ and infant Q-CHAT) were assessed for extreme outliers, as defined by an interval of three times the interquartile range. If present, these were reduced to the highest possible value within the interval to facilitate statistical testing.

All steroid hormone concentration values exhibited varying degrees of positive skew and were log-transformed to reduce this and facilitate subsequent statistical testing via linear regression. Following this, outliers were preserved in the analysis to retain clinical heterogeneity. Human choriogonadotropin (hCG) and pregnancy-associated peptide alpha (PAPP-A) values were retrieved from participants’ clinical records. These had been standardised according to multiple of the median (MoM) by the Prenatal Screening Department of the Trust according to maternal age, gestational age, and the national means [46, 54].

Multivariate analysis was conducted by calculating composite scores for the free fractions of estradiol (E2) and testosterone (T), and by estimating overall steroidogenesis. The free testosterone index (FTI) and free estradiol index (FEI) were calculated via the following formulas (all concentrations in nmol/L) and then log-transformed for further statistical analysis, as previously suggested [45]:

$$\begin{aligned} &{{FEI:}}\,(100*[E2])/([SHBG])\\ &{{FTI:}}\,(100*[T])/([SHBG]) \\ \end{aligned}$$

Latent factor analysis (‘nFactors’ package) was used to identify the optimal number of common steroidogenic factors based on their correlation matrix. Values for the predicted steroidogenic factor were calculated for each individual via the “Bartlett” method, based on the predicted loadings.

A series of clinical characteristics and group covariates were assessed for pairwise association with autistic traits. In cases of binary traits, differences were tested via Student’s *t*-tests. These included comorbidity with PCOS or family history of autism, the latter being defined as present if the participating mothers reported having a first-degree relative (including previous child) that had been diagnosed with autism. A score of clinical severity of maternal hirsutism was devised based on responses in the Pregnancy History Questionnaire (PHQ), which the participating mothers completed following labour. A score of 1 denoted selection of one area of excess hair growth and 2 denoted more than one area. These were further treated as group variables (i.e. “no hirsutism”, “one area”, “more than one area”) and were used in cohort comparisons in terms of hormone levels and autistic traits.

Circulating hormones were log-transformed and assessed for association with autistic traits. For AQ, pairwise Pearson’s correlations were first used and then followed-up with a linear regression model with AQ as the outcome variable and the following predictor variables: hormonal concentration, maternal age, comorbidity with PCOS. To account for potential underlying associations between infant sex and Q-CHAT scores, only multiple regression models were used in which an interaction term between infant sex and hormonal concentration was added (for each hormone separately), with Q-CHAT scores as the outcome, and addition of the following covariates: maternal age, maternal PCOS, maternal AQ scores, and infant age at assessment adjusted for gestational age at birth. Family history of autism was not included as a covariate in these models, given the very small number of mothers with a diagnosed first-degree relative who also had available serum for hormone assays (*n* = 1 overlap).

The same approach and models were used when examining the hormone composite measures, namely FEI, FTI and the factors defined by unsupervised factor analysis of the steroid hormones.

To ensure their validity, multiple regression models with significant findings for AQ or Q-CHAT were further tested for heteroscedasticity via the studentized Breusch–Pagan test and for the non-normality of their residuals via the Shapiro–Wilk test (Additional file 1: Table S8).

## Results

### Cohort characteristics and autistic traits

Of the *n *= 219 pregnant women who consented to take part in the study, *n *= 17 had a first-degree relative with autism, *n *= 26 had been diagnosed with PCOS, and *n *= 89 responded positively to having excess body hair growth in the past. Overall mean age of the mothers was 32.4 years (SD = 4.54). Of this cohort, *n *= 189 completed the Autism Spectrum Quotient (AQ), with scores ranging from 1 to 47 (mean = 14.63, SD = 8.11) (Fig. 1).

**Fig. 2:**
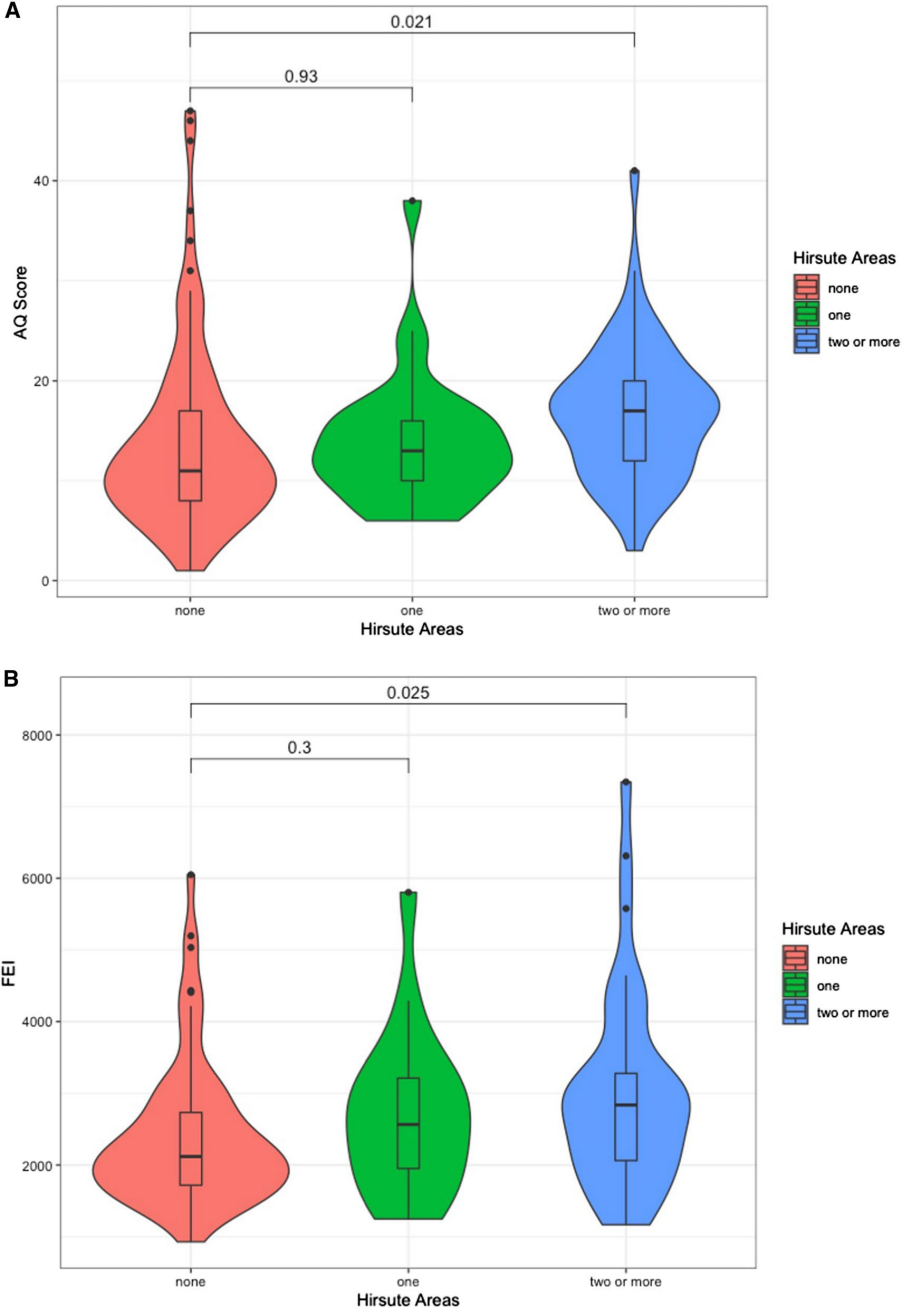
Violin-boxplots showing the distribution of **A** maternal autistic traits (AQ) and **B** free estradiol levels (FEI), according to clinical history of hirsutism. Women that reported two or more body areas affected had significantly higher levels of both AQ (*p* = 0.021) and FEI (*p* = 0.025)

Women with a family history of autism had a significantly higher AQ score [*n *= 13, mean = 28 (SD = 14.36)] compared to women without any first-degree relatives with autism [*n *= 176, mean = 13.64 (SD = 6.51)] (Cohen’s *D* = 1.98, *p* = 0.004) (Table 1). Of the women who replied to both questionnaires, those who reported excess body hair in more than one area of their body (*n *= 51) also had significantly higher AQ scores [mean = 16.8 (SD = 6.7)], than those without any sign of excess body hair growth [*n *= 109, mean = 13.8 (SD = 8.9)] (Cohen’s *D* = 0.36, *p* = 0.021) (Fig. 2A). This effect persisted after controlling for family history of autism, maternal age and a diagnosis of PCOS via a linear regression model [*Beta* = 1.9 (SE = 0.67), semipartial *r* = 0.18, *p* = 0.005] (Additional file 1: Table S2).

**Table 1:** Cohort characteristics and associations between clinical/demographic factors and autistic traits

Categorical	AQ				Q-CHAT			
	*N*	Mean traits (SD)	Cohen’s *D*	*p*	*N*	Mean traits (SD)	Cohen’s *D*	*p*
Family history of autism	13 with	28.00 (14.4)	***D* = 1.98**	*p* = 0.0036**	14 with	31.00 (11.0)	*D* = 0.14	*p* = 0.720
	176 without	13.64 (6.5)			165 without	29.90 (7.5)		
Maternal PCOS	24 with	14.96 (6.7)	*D* = 0.05	*p* = 0.804	22 with	27.86 (6.3)	*D* = 0.31	*p* = 0.115
	165 without	14.58 (8.3)			157 without	30.28 (8.0)		
Fetal sex	87 males	14.15 (8.1)	*D* = 0.11	*p* = 0.453	87 males	31.00 (8.1)	*D* = 0.09	*p* = 0.540
	102 females	15.04 (8.2)			92 females	29.63 (7.6)		
History of Hirsutism	109 none	13.8 (8.0)			102 none	29.27 (7.8)		
	29 in one area	13.9 (6.9)	*D* = 0.01	*p* = 0.930	28 in one area	31.5 (7.5)	*D* = 0.28	*p* = 0.181
	51 in two + areas	16.8 (6.9)	D = 0.36	*p* = 0.021*	49 in two + areas	30.63 (8.0)	*D* = 0.17	*p* = 0.326

Continuous	*N*	Mean factor	Pearson’s *r*	*p*	*N*	Mean factor	Pearson’s *r*	*p*
Maternal age	154	32.50 (4.5) years	*r* = −0.05	*p* = 0.462	135	32.60 (4.5) years	*r* = −0.14	*p* = 0.065
Birth weight	164	3399.5 (516.6) g	*r* = −0.04	*p* = 0.596	157	3402.8 (509.8) g	*r* = −0.03	*p* = 0.727
Infant age-adjusted					148	572.9 (23.8) days	*r* = −0.09	*p* = 0.250

Test coefficients are Cohen’s *D* and Pearson’s *r* correlation coefficient. *: *p *< 0.05, **: *p* < 0.01, ***: *p* < 0.001

Of these pregnancies, *n *= 178 of the infants were followed-up with the Q-CHAT when they were older than 18 months of age (range 541–671 days after birth), with most being assessed between 18 and 20 months (mean = 570 days, SD = 21.3 days) (Fig. 1). Prior to statistical analysis, infant age was adjusted according to the gestational age at birth for each infant (mean = 39.56, SD = 1.5 weeks post-conception).

Regarding Q-CHAT scores, one extreme outlier was noted in the distribution (Q-CHAT = 71), which was reduced to the highest value within an interval of three times the interquartile range (Q-CHAT = 53), in order to reduce skewness and facilitate statistical comparisons.

There was no significant difference between Q-CHAT scores of male [mean = 30.35 (SD = 8.13)] and female [mean = 29.63 (SD = 7.58)] infants at this time-point of assessment (Cohen’s *D* = 0.09, *p* = 0.54).

Q-CHAT scores were significantly correlated with maternal AQ scores (Pearson’s *r* = 0.21, *p* = 0.008) (Additional file 1: Fig. S1).

### Hormone covariates and factor analysis

Hormone concentrations were only available in subsets of the cohort of women that consented to the study. This differed slightly for placental markers that were part of routine prenatal screening (hCG and PAPP-A) and steroid level measurements, which were analysed for research purposes on the remaining serum sample for each participant (Fig. 1).

**Fig. 3:**
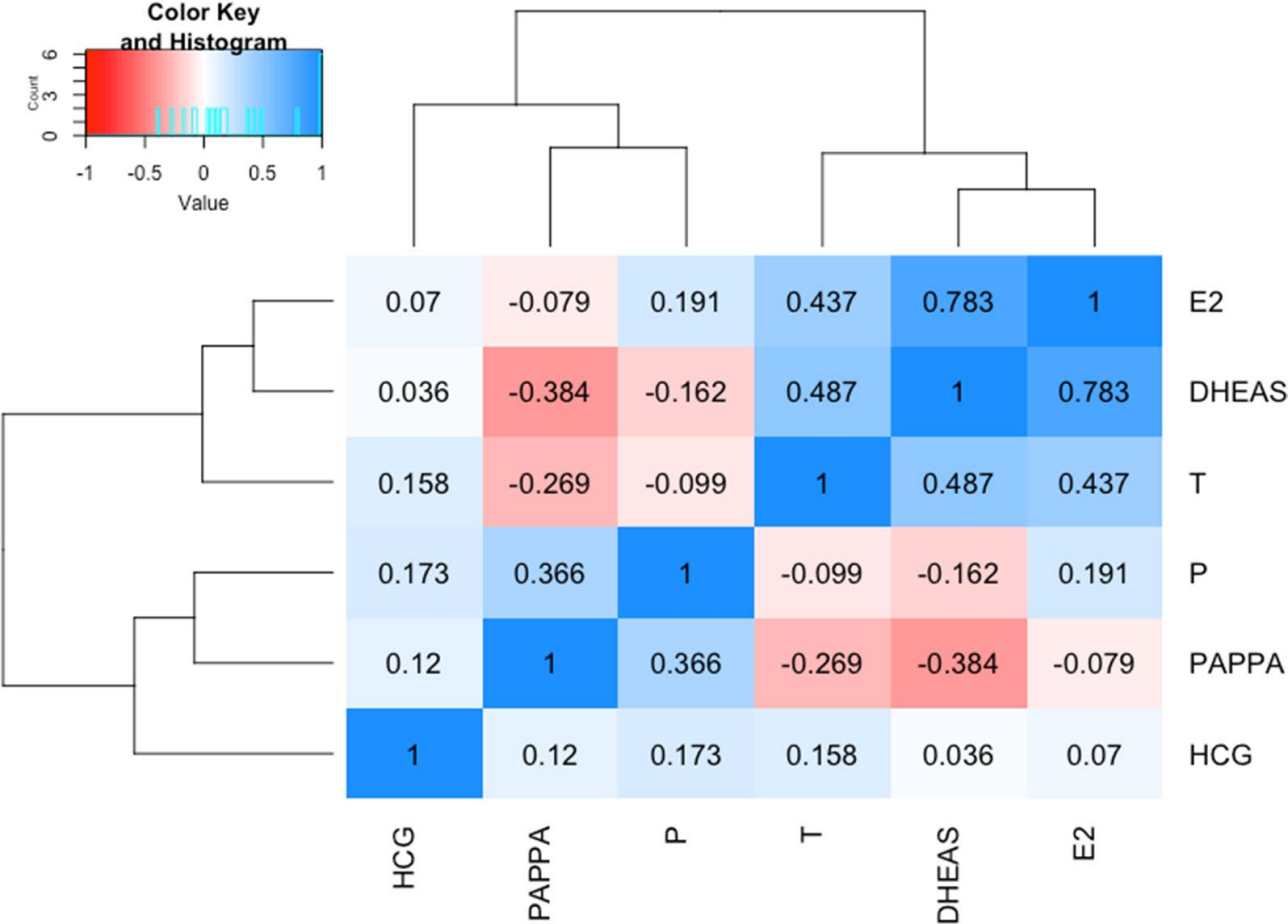
Heatmap and dendrogram of the pairwise correlations of the log-transformed hormone concentrations. Values indicate the Pearson's correlation coefficients

The analysed maternal serum samples corresponded to a narrow period of gestation between the late first and early second trimester (mean = 12.7 weeks, SD = 0.8 weeks). Circulating hormones showed varying degrees of correlation with each other (Fig. 3) and with other demographic and clinical variables. Testosterone, DHEAS and progesterone were all positively correlated with maternal age (Additional file 1: Table S3). Unsupervised factor analysis showed that a common latent factor could be derived from estradiol, testosterone and DHEAS, which account for 32% of the total variance in hormone levels. The values for this ‘steroidogenic factor’ were estimated for each participant based on the factor loadings and steroid concentrations (Additional file 1: Figure S2).

Women with PCOS also had significantly higher levels of estradiol and progesterone, but lower levels of SHBG, compared to women without the condition (Additional file 1: Table S4). Maternal age correlated positively with progesterone, but negatively with testosterone, FTI and DHEAS (Additional file 1: Table S4). Women with a history of hirsutism in more than one area of their body also had significantly higher levels of estradiol (Cohen’s *D* = 0.47, *p* = 0.034) and FEI (Cohen’s *D* = 0.51, *p* = 0.025), as well as higher predicted values for the latent steroid factor (*D* = 0.50, *p* = 0.028) (Fig. 2B).

### Associations between hormones and maternal AQ score

**Fig. 4:**
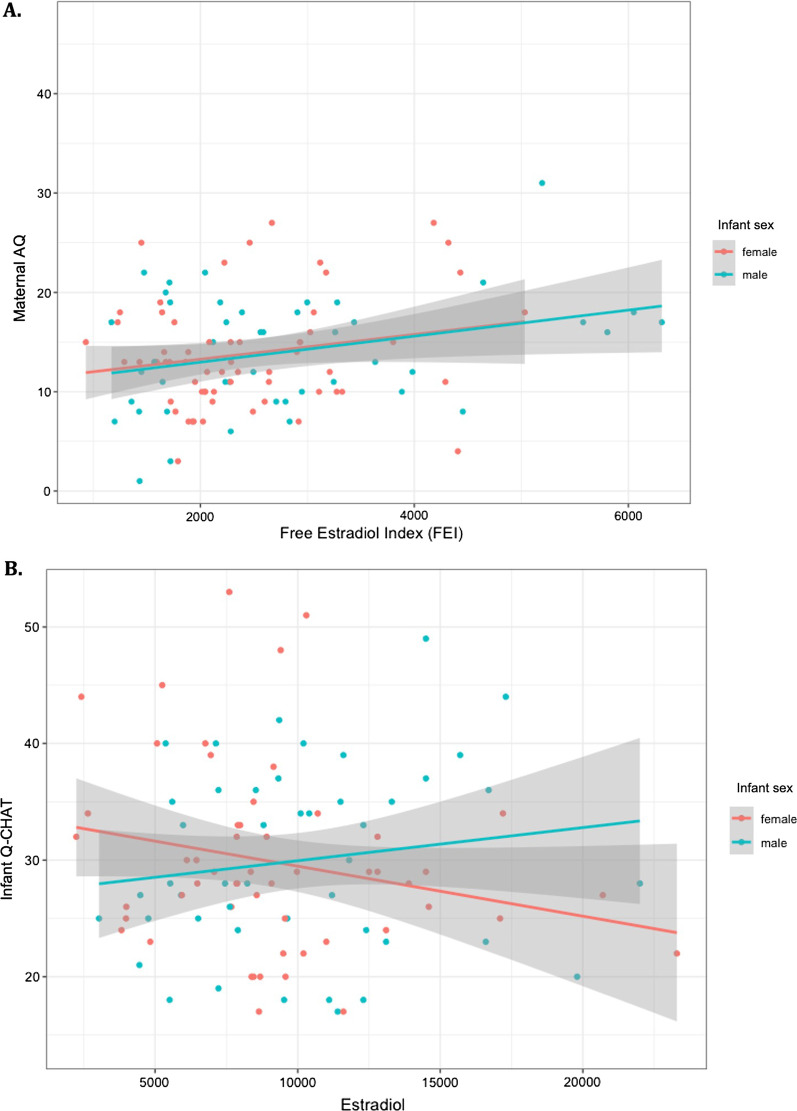
Scatterplots with linear fit-models for **A** the association between maternal free estradiol (FEI) and AQ score, and **B** the association between maternal estradiol and Q-CHAT; separate linear models are presented for each sex and show significant interactions with infant sex in the case of infant Q-CHAT

The association between hormones in maternal serum and maternal autistic traits was investigated via univariate Pearson’s correlation coefficient and with multiple regression (MR) controlled for maternal age and a diagnosis of PCOS (Table 2). FEI was significantly correlated with maternal AQ score using both methods (MR: semipartial *r* = 0.19, *p* = 0.048) (Fig. 4A). Estradiol levels were associated with AQ in Pearson’s correlation (*r* = 0.20, *p* = 0.036), but this was not statistically significant in the multiple regression model that controlled for other covariates (MR: semipartial *r* = 0.11, *p* = 0.25).

**Table 2:** Pearson’s correlation (hormone and AQ) and multiple regression models (MR) for predicting AQ scores, controlled for maternal age and a diagnosis of PCOS

	Test coefficient	SE	Semipartial correlation	*p* value
*Estradiol*				
Pearson’s	*r* = 0.20	0.09		*p* = 0.036*
MR: intercept	*Beta* = 4.52	12.29		*p* = 0.714
Predictor	*Beta* = 1.43	1.23	*r* = 0.11	*p* = 0.250
*Testosterone*				
Pearson’s	*r* = 0.09	0.10		*p* = 0.377
MR: intercept	*Beta* = 17.34	4.50		*p* < 0.001***
Predictor	*Beta* = 0.34	1.12	*r* = 0.03	*p* = 0.761
*DHEAS*				
Pearson’s	*r* = 0.09	0.10		*p* = 0.379
MR: intercept	*Beta* = 15.29	6.47		*p* = 0.020*
Predictor	*Beta* = 0.44	0.86	*r* = 0.05	*p* = 0.600
*Progesterone*				
Pearson’s	*r* = − 0.09	0.09		*p* = 0.365
MR^a^: intercept	*Beta* = 23.23	6.94		*p* = 0.001**
Predictor	*Beta* = −2.16	2.22	*r* = −0.09	*p* = 0.334
*hCG MoM*				
Pearson’s	*r* = −0.03	0.08		*p* = 0.665
MR: intercept	*Beta* = 13.92	0.97		*p* < 0.001***
Predictor	*Beta* = −0.27	0.58	*r* = −0.04	*p* = 0.650
*PAPP-A MoM*				
Pearson’s	*r* = −0.04	0.08		*p* = 0.589
MR : intercept	*Beta* = 14.18	1.16		*p* < 0.001***
Predictor	*Beta* = −0.49	0.84	*r* = −0.05	*p* = 0.560
***Composite measures***				
*Free Estradiol Index*				
Pearson’s	*r* = 0.22	0.09		*p* = 0.019*
MR: intercept	*Beta* = 13.76	4.61		*p* = 0.004**
Predictor	*Beta* = 2.76	1.4	*r* = 0.19	*p* = 0.048*
*Free Testosterone Index*				
Pearson’s	*r* = 0.11	0.10		*p* = 0.258
MR: intercept	*Beta* = 17.48	4.21		*p* < 0.001
Predictor	*Beta* = 0.69	4.21	*r* = 0.07	*p* = 0.484
*Steroid factor*				
Pearson’s	*r* = 0.18	0.10		*p* = 0.061
MR: intercept	*Beta* = 16.75	0.09		*p* < 0.001***
Predictor	*Beta* = 0.77	0.55	*r* = 0.17	*p* = 0.085

*: *p* < 0.05, **: *p* < 0.01, ***: *p* < 0.001

### Associations between hormones and infant Q-CHAT scores

Log-transformed concentrations of hormone levels were studied in relation to infant Q-CHAT with multiple linear regression models that controlled for birth weight, maternal PCOS, maternal AQ and infant age at the time of Q-CHAT, adjusted for gestational age at birth. The interaction between hormone concentration and infant sex was also used as a variable in the same model (Table 3). Infant sex moderated the association between maternal estradiol levels and Q-CHAT scores, with a positive association for Q-CHAT in males but not females (hormone-by-sex: semipartial *r* = 0.23, *p* = 0.036) (Table 3; Fig. 4B).

**Table 3:** Multiple regression models for predicting Q-CHAT scores and accounting for a hormone-by-sex interaction, further controlled for maternal AQ, a diagnosis of PCOS, maternal age, birth weight and infant age at Q-CHAT assessment-adjusted for gestational age at birth

	Q-CHAT			
	Regression coefficient	SE	Semipartial correlation coefficient	*p* value
*Estradiol*				
MR: intercept	*Beta* = 114.20	35.21		*p* = 0.002**
Hormone	*Beta* = −4.00	2.311	− 0.19	*p* = 0.087
Hormone-by-sex	*Beta* = 8.27	4.08	0.23	*p* = 0.036*
*Testosterone*				
MR: intercept	*Beta* = 80.01	29.07		*p* = 0.007**
Hormone	*Beta* = −1.95	2.12	− 0.10	*p* = 0.362
Hormone-by-sex	*Beta* = 0.99	3.28	0.03	*p* = 0.765
*DHEAS*				
MR: intercept	*Beta* = 90.03	29.68		*p* = 0.003**
Hormone	*Beta* = −2.33	1.58	− 0.16	*p* = 0.144
Hormone-by-sex	*Beta* = 1.36	3.22	0.04	*p* = 0.674
*Progesterone*				
MR : intercept	*Beta* = 84.06	32.16		*p* = 0.011*
Hormone	*Beta* = −3.70	4.65	− 0.08	*p* = 0.429
Hormone-by-sex	*Beta* = 11.64	6.86	0.18	*p* = 0.095
*hCG MoM*				
MR: intercept	*Beta* = 34.2	20.61		*p* = 0.100
Hormone	*Beta* = 2.95	1.07	0.24	*p*= 0.007**
Hormone-by-sex	*Beta* = −4.34	1.53	− 0.25	*p* = 0.005**
*PAPP-A MoM*				
MR: intercept	*Beta* = 46.04	21.13		*p* = 0.031*
Hormone	*Beta* = 1.69	1.45	0.11	*p* = 0.245
Hormone-by-sex	*Beta* = −1.37	2.29	− 0.05	*p* = 0.552
***Composite measures***				
*Free Estradiol Index*				
MR: intercept	*Beta* = 82.42	29.02		*p* = 0.006**
Hormone	*Beta* = −0.969	1.37	− 0.07	*p* = 0.481
Hormone-by-sex	*Beta* = 1.989	1.68	0.13	*p* = 0.240
*Free Testosterone Index*				
MR: intercept	*Beta* = 77.86	29.16		*p* = 0.009**
Hormone	*Beta* = 2.50	8.78	*r* = 0.03	*p* = 0.777
Hormone-by-sex	*Beta* = −9.53	11.69	*r* = −0.09	*p* = 0.417
*Steroid factor*				
MR: intercept	*Beta* = 85.95	29.01		*p* = 0.004**
Hormone	*Beta* = −1.95	1.20	*r* = −0.17	*p* = 0.108
Hormone-by-sex	*Beta* = 2.80	1.81	*r* = 0.16	*p* = 0.126

*: *p* < 0.05, **: *p* < 0.01, ***: *p* < 0.001

Standardised hCG MoM values were significantly associated with Q-CHAT scores, with a significant moderating effect for infant sex (hormone-by-sex: semipartial *r* = −0.26, *p* = 0.005). This effect followed the opposite pattern to estradiol, with a positive relationship in females but not males (Table 3).

All multiple regression models that yielded significant results (for both AQ and Q-CHAT) passed tests on the assumptions of homoscedasticity and normality of residuals (full model results: Additional file 1: Tables S6–S8).

## Discussion

This is the first clinical longitudinal study to report on the endocrine profile of pregnant women and how this relates to their own autistic traits and to the autistic traits of their infants. First, we found that the fraction of free circulating estradiol (FEI) during pregnancy correlated positively with the autistic traits of pregnant neurotypical women. Second, we found that maternal estradiol was associated with infant autistic traits (as measured by the Q-CHAT) in a sex-dependent way, with a positive correlation in males but not females. Third, we found that the opposite was true for standardised hCG MoM values, where a negative correlation with Q-CHAT scores in males was noted. These associations with early neurodevelopment were independent of the mother’s AQ score or PCOS status, as well as other infant characteristics such as birth weight or age at the time of assessment. Finally, this is the first longitudinal study to show a significant positive correlation between maternal and infant autistic traits, as measured by the AQ and Q-CHAT, respectively.

The association between free circulating estradiol and maternal AQ was independent of maternal age or a diagnosis of PCOS. In addition, women with clinical history of excess body hair growth in more than one area of their body also had significantly higher autistic traits scores, as well as higher levels of circulating estradiol and FEI (Fig. 2). This is consistent with previous studies that reported higher rates of steroid-exposure related symptoms, such as hirsutism in autistic women [40]. The correlation between FEI and AQ was independent of PCOS diagnostic status, and women diagnosed with PCOS did not have higher AQ scores (Table 1). This may be due to reduced statistical power (*n *= 13 with PCOS diagnosis) or the fact that all the women in this cohort had no autism diagnosis themselves and were without significant challenges in fertility, contrary to previous studies that found a link between PCOS and autism [17].

There was no association between testosterone levels or the FTI and maternal autistic traits in this study. Testosterone correlated positively with estradiol (Additional file 1: Table S3) but did not differ between women with and without PCOS, or correlate with a clinical history of hirsutism. Previous studies have also shown that circulating testosterone levels measured at a single time-point do not always correlate closely with their associated clinical parameters, such as hair growth [39]. Particularly during pregnancy, estradiol levels may be more clinically informative, as testosterone is rapidly aromatised into estradiol by the placenta. Estradiol could then be interpreted as the end-product of wider steroidogenesis and may be a better biomarker of the ‘steroidopathy’ previously indicated by epidemiological studies of autistic women [40].

This is also the first study to investigate circulating hormones in maternal serum in relation to autistic traits in their infants measured between 18 and 20 months of age via the Q-CHAT. This questionnaire was developed to enable parents to quantify autistic traits, moving away from a restrictive binary format of items toward a Likert format. To date, the Q-CHAT has been validated in several studies. Interestingly, the Q-CHAT also shows sex differences in early autistic traits, with males scoring significantly higher than females in larger cohorts [2, 3, 41, 43].

The current study found significant moderation effects of infant sex on the associations between hormones and neurodevelopment. Specifically, increased maternal estradiol was more predictive of Q-CHAT scores of males than females (Fig. 4; Table 3). This was independent of potential confounding variables, such as maternal age, diagnosis of PCOS and maternal autistic traits. Contrary to the results with the AQ, free estradiol levels (FEI), as estimated via serum SHBG, were not predictive of Q-CHAT scores. SHBG is a peptide, and it does not cross the placenta as easily as steroids, as shown in rare cases of partial deficiency in mothers but not their fetus [26]. Maternal SHBG may therefore not accurately capture the bioavailability of steroids in the fetal circulation nor accurately predict potential effects on infant neurodevelopment.

Further research is needed to understand the interaction between maternal estradiol levels and fetal sex in predicting Q-CHAT scores. As with the observed male bias in autism diagnoses, the lack of an association between hormone levels in females may reflect behavioural differences that are not adequately captured by this particular instrument [32]. Alternatively, this interaction may represent evidence of differential liability to prenatal hormone exposure, whereby males are affected more than females by the same maternal endocrine factors. Since males are undergoing an additional increase in steroid levels during mid-pregnancy, due to the activation of the testes, this interaction could also be attributed to the added effect of elevated steroids in the fetal circulation of males [51]. While fetal steroids were not measured directly in this study, previous comparisons in humans showed that estradiol levels correlated between maternal serum and amniotic fluid, in both the second and third trimesters [50]. The same was found for hCG levels in the second trimester [47]. In contrast, increases in androgen levels of male pregnancies were not detectable in maternal serum [50]. Therefore, the interaction of sex with estradiol levels in predicting Q-CHAT scores, in this study, could potentially be attributed to the additive effect of elevated androgens in the fetal circulation of males, which may not have been captured by assaying maternal serum, rather than amniotic fluid [5].

The findings with hCG mirror those for estradiol, showing a negative association with Q-CHAT scores in females but not males. hCG is produced by the developing trophoblast cells in the placenta and regulates early implantation as well as steroid production [14]. hCG levels also show baseline sex differences in typical cohorts as early as the first half of pregnancy [1]. In the current cohort, hCG was measured late in the first trimester, during the first ultrasound visit, as part of the screening programme for Down’s syndrome. This is part of routine prenatal screening in the UK to test for placental dysfunction that can often be indicative of genomic instability due to aneuploidies [54]. In cases of clinically diagnosed autism, both very low and very high levels of hCG have been found in maternal serum, leading to a “U-shaped” association when studying both males and females [53]. Furthermore, autistic traits in the children have been associated with the severity of nausea and ‘morning sickness’ during pregnancy, symptoms that have also been linked to high hCG levels [22, 52]. Further studies into placental functionality could offer insight into these observations, the role of sex, and more specifically, whether the observed interaction effect for Q-CHAT score is part of an adaptive response that is more pronounced in females than males.

## Limitations

Limitations of the current study include the relatively small sample size, as well as potential ascertainment bias given the voluntary process of recruitment. In addition, AQ and Q-CHAT scores were both rated by the mother herself. This was addressed by controlling for maternal AQ when studying associations between hormones and infant Q-CHAT scores. The items of the AQ-Adult and Q-CHAT are also substantially different, with the latter dealing with behavioural and developmental milestones that are specific to infants rather than to interests and personality traits that are more evident in adulthood. Furthermore, the findings of the current study may have been inflated by Type I errors, as *p* value thresholds were not corrected for multiple testing. However, the findings on estradiol were consistent for both maternal and infant outcomes (Fig. 4), were reflected in clinical hirsutism differences (Fig. 2), and are in accordance with those observed in other studies of clinical autism [10, 13]. The high degree of correlation between many of the assessed hormones (Fig. 3) also indicates a common functional and regulatory framework. The association tests for individual hormones may therefore not be entirely independent, but instead affected by a common steroidogenic factor as previously reported [9]. Replication of these findings in a larger, independent cohort is warranted to confirm their validity.

## Conclusions

This is the first longitudinal study to report associations between maternal steroidogenic factors and the autistic traits of both the mother and her infant, with significant moderating effects of sex being noted for the latter. Additional research is needed to replicate these findings, to establish how maternal steroidogenesis may affect fetal neurodevelopment, and to determine how these processes interact with genetics to disproportionately increase the liability for autism in males.

## Supplementary Information

**Additional file 1.** Supplementary Figures and Tables.

## Data Availability

The datasets generated during and/or analysed during the current study are not publicly available due to limited ethics approval for the wider clinical study (CUSP) by CUH and to the specific consent provided by the participants. They may be available from the corresponding author on reasonable request and pending approval of any future analyses by CUH.

## Abbreviations

AQ: Autism Spectrum Quotient
BDNF: Brain-derived neurotrophic factor
CBAL: Core Biochemical Assays Laboratory
CUSP: Cambridge Ultrasound Siblings and Parents Project
DHEAS: Dehydroepiandrosterone sulphate
E2: Estradiol
FEI: Free Estradiol Index
FTI: Free Testosterone Index
hCG: Human chorionic gonadotropin
IUGR: Intrauterine growth restriction
LGA: Large for gestational age
MoM: Multiple of the median
P: Progesterone
PAPP-A: Pregnancy-associated peptide alpha
PCOS: Polycystic ovary syndrome
PHQ: Pregnancy History Questionnaire
Q-CHAT: Quantitative Checklist of Autism in Toddlers
SD: Standard deviation
SHBG: Sex hormone binding globulin
