# Supplementary material for: Maternal steroid levels and the autistic traits of the mother and infant
Source: Mol Autism. 2021 Jul 8;12:51. doi: 10.1186/s13229-021-00453-7 (PMC8268382; doi:10.1186/s13229-021-00453-7)
Supplement: Supplementary file 1 — Additional file 1. Supplementary Figures and Tables. [file 13229_2021_453_MOESM1_ESM.docx]

### **Supplementary Tables & Figures**

|  | Chemiluminescence Immunoassay Assays by DiaSorin | Units of Measurement | Lower limit of detection | Imprecision In-House Measurements |
| --- | --- | --- | --- | --- |
| Estradiol | Estradiol II Gen No.310680  (issued 6-2016) | pmol/L | 36.7 | 2.9% at 425 pmol/L, & 1.9% at 1223 pmol/L |
| Testosterone | Testosterone No.310410  (issued 12-2014) | nmol/L | 0.17 | 4.9% at 6.37 nmol/L, & 4.4% at 18.2 nmol/L |
| DHEAS | DHEA-S, No.310430, (issued 04-2016) | ug/L | 1.0 | 4.9% at 6.4 ųg/L, &  4.4% at 18.2 ųg/L |
| Progesterone | Progesterone II Gen No.310690, (issued 06-2016) | ng/L | 0.12 | 4.2% at 2.2 ng/L, &  2.5% at 21.4 ng/L |
| SHBG | SHBG, No. 319020, (issued 03-2017) | nmol/L | 0.2 | 9.9% at 43 nmol/L, & 7.2% at 190 nmol/L |

***Suppl Table 1****: Assay details, as conducted for research purposes by the Core Biochemical Assay Laboratory (CBAL) of CUH NHS Foundation Trust.*


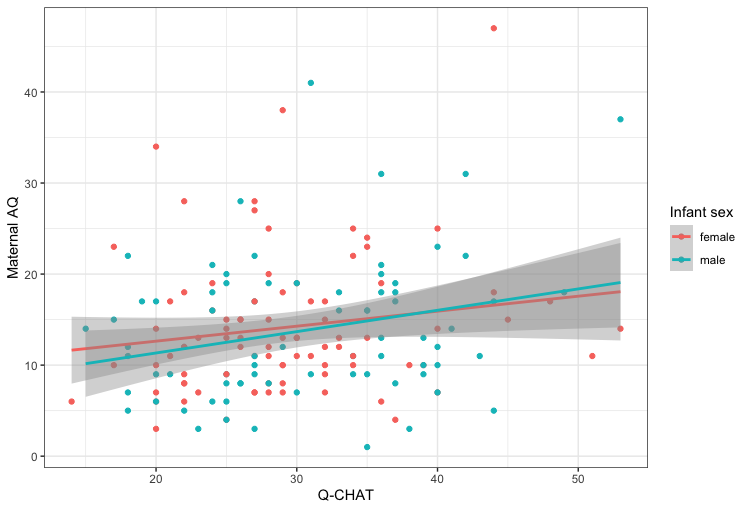


***Suppl Figure 1***: Maternal AQ and Q-CHAT of the same mother-infant pairs, showing a high degree of correlation between them.

|  | Coefficient | SE | Semipartial | p-value |
| --- | --- | --- | --- | --- |
| Intercept | 20.36 | 3.86 |  | <0.0001 |
| Hirsutism | 1.91 | 0.67 | 0.18 | 0.005 |
| PCOS | -0.35 | 2.69 | -0.01 | 0.88 |
| Hirs*PCOS | 0.32 | 1.78 | 0.01 | 0.86 |
| Maternal Age | -0.25 | 0.12 | -0.14 | 0.03 |
| Autism in Family | 15.61 | 2.1 | 0.48 | <0.0001 |
|  |  |  | **Adjusted R2**=0.24 | **Model p**<0.0001 |

***Suppl Table 2****:* Multiple regression model of the associations between maternal hirsutism score and AQ.

|  | DHEAS | Progesterone | Estradiol | Testosterone | hCG | PAPP-A |
| --- | --- | --- | --- | --- | --- | --- |
| DHEAS | 1 |  |  |  |  |  |
| Progesterone | -0.214* | 1 |  |  |  |  |
| Estradiol | 0.655*** | 0.199* | 1 |  |  |  |
| Testosterone | 0.540*** | -0.037 | 0.51*** | 1 |  |  |
| hCG MoM | 0.019 | 0.179 | 0.101 | 0.214* | 1 |  |
| PAPP-A MoM | -0.36*** | 0.380*** | -0.041 | -0.159 | 0.048 | 1 |

**Suppl Table 3**: Pairwise correlation coefficients (Pearson’s r) for each pair of prenatal factors, with asterisks denoting statistical significance.


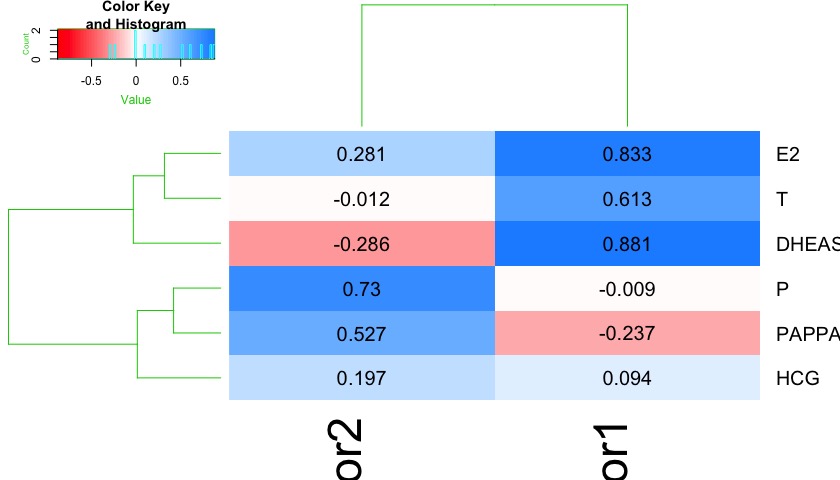


**Suppl. Figure 2**: Heatmap and dendrogram of the loadings of each of the tested variables on latent factors. Factor 1 includes all sex steroids measured in the current study

|  | Maternal PCOS | | Hirsutism Score  (0 to 2+ areas) | | Maternal Age | |
| --- | --- | --- | --- | --- | --- | --- |
|  | *Effect size* | *p* | *Effect size* | *p* | *Effect size* | *p* |
| Estradiol | **D=1.06** | **0.008** | **D=0.47** | **0.034** | r=-0.1 | 0.274 |
| Testosterone | D=0.35 | 0.247 | D=0.21 | 0.328 | **r=-0.37** | **<0.001** |
| DHEAS | D=0.13 | 0.54 | D=0.33 | 0.127 | **r=-0.30** | **<0.001** |
| Progesterone | **D=0.92** | **0.018** | D=0.001 | 0.995 | **r=0.31** | **<0.001** |
| SHBG | **D=0.62** | **0.017** | D=0.03 | 0.870 | r=0.06 | 0.51 |
| FEI | D=0.58 | 0.100 | **D=0.51** | **0.025** | r=-0.17 | 0.08 |
| FTI | D=0.03 | 0.888 | D=0.25 | 0.268 | **r=-0.38** | **<0.001** |
| Steroid Factor | D=0.50 | 0.135 | **D=0.50** | **0.028** | **r=-0.22** | **0.015** |
| hCG MoM | D=0.07 | 0.762 | D=0.11 | 0.542 | r=-0.04 | 0.61 |
| PAPP-A MoM | D=0.18 | 0.291 | **D=0.38** | **0.016** | r=0.08 | 0.261 |

***Suppl Table 4****: Associations between circulating hormones/peptides and maternal factors*

|  | Males |  | Females |  |  |  |
| --- | --- | --- | --- | --- | --- | --- |
|  | *Mean* | *SD* | *Mean* | *SD* | *Coeff* | *p* |
| Q-CHAT | 30.36 | 8.13 | 29.63 | 7.58 | -0.62 | 0.538 |
| Age at follow-up | 575.49 | 21.54 | 570.60 | 25.65 | -1.26 | 0.210 |
| Birth Weight | 3461.8 | 481.49 | 3363.35 | 538.99 | -1.34 | 0.183 |
| Maternal age | 32.68 | 4.83 | 32.16 | 4.27 | -0.85 | 0.396 |
| Maternal AQ | 14.15 | 8.06 | 15.04 | 8.18 | 0.751 | 0.453 |
|  |  |  |  |  |  |  |
|  | **Males** |  | **Females** |  |  |  |
|  | *N - with* | *N - without* | *N - with* | *N - without* | *Coeff* | *p* |
| PCOS | 17 | 87 | 9 | 106 | 3.02 | 0.082 |
| Autism in Family | 9 | 95 | 8 | 107 | 0.05 | 0.83 |

***Suppl Table 5****: Sex differences among infant clinical characteristics*

|  | Coefficient | SE | Semipartial | p-value |
| --- | --- | --- | --- | --- |
| Intercept | 13.77 | 4.60 |  | 0.003 |
| FEI | 2.76 | 1.40 | 0.19 | 0.048 |
| maternal age | -0.07 | 0.13 | -0.05 | 0.567 |
| PCOS | 1.14 | 1.68 | 0.06 | 0.498 |
|  |  |  | **Adjusted R2**=0.03 | **Model p=**0.102 |

***Suppl Table 6****: Full model results of the association of FEI to maternal AQ*

|  | Coefficient | SE | Semipartial | p-value |
| --- | --- | --- | --- | --- |
| Intercept | 114.21 | 35.21 |  | 0.002 |
| Estradiol (E2) | -4.00 | 2.31 | -0.19 | 0.087 |
| Sex | -72.96 | 37.27 | -0.22 | 0.054 |
| E2 * Sex | 8.27 | 4.08 | 0.23 | 0.036 |
| Infant Age | -0.04 | 0.04 | -0.12 | 0.287 |
| Birth Weight | -0.002 | 0.002 | -0.11 | 0.347 |
| Maternal Age | -0.59 | 0.04 | -0.30 | 0.007 |
| Maternal AQ | 0.10 | 0.16 | 0.07 | 0.544 |
| PCOS | -2.04 | 2.83 | -0.08 | 0.473 |
|  |  |  | **Adjusted R2**=0.09 | **Model p=**0.052 |

***Suppl Table 7****: Full model results of the association of estradiol (E2) to infant Q-CHAT*

|  | AQ | | Q-CHAT | |
| --- | --- | --- | --- | --- |
|  | Normality of Residuals | Homoscedasticity  (Breusch-Pagan) | Normality of Residuals  (Shapiro-Wilk) | Homoscedasticity  (Breusch-Pagan) |
| Estradiol | | | | |
| Test statistic | W= 0.98 | BP=1.19 | W=0.98 | BP=0.95 |
| p-value | p=0.175 | p=0.755 | p=0.152 | p=0.996 |
| Testosterone | | | | |
| Test statistic | W=0.98 | BP=0.93 | W=0.98 | BP=12.8 |
| p-value | p=0.105 | p=0.817 | p=0.154 | p=0.119 |
| DHEAS | | | | |
| Test statistic | W=0.98 | BP=1.09 | W=0.98 | BP=14.14 |
| p-value | p=0.105 | p=0.78 | p=0.213 | p=0.08 |
| Progesterone | | | | |
| Test statistic | W=0.98 | BP=0.55 | W=0.97 | BP=9.80 |
| p-value | p=0.09 | p=0.91 | p=0.05 | p=0.27 |
| hCG MoM | | | | |
| Test statistic | **W=0.96** | BP=0.85 | W=0.98 | BP=10.58 |
| p-value | **p=0.0003** | p=0.65 | p=0.167 | p=0.226 |
| PAPP-A MoM | | | | |
| Test statistic | **W=0.96** | BP=0.86 | W=0.98 | BP=12.36 |
| p-value | **p=0.0004** | p=0.65 | p=0.104 | p=0.136 |
|  | | | | |
| Composite scores | | | | |
| Free Estradiol Index | | | | |
| Test statistic | W=0.98 | BP=1.54 | W=0.98 | BP=13.22 |
| p-value | p=0.142 | p=0.673 | p=0.491 | p=0.104 |
| Free Testosterone Index | | | | |
| Test statistic | W=0.98 | BP=0.89 | W=0.98 | BP=11.72 |
| p-value | p=0.09 | p=0.828 | p=0.129 | p=0.164 |
| Steroid Factor | | | | |
| Test statistic | W=0.98 | BP=0.58 | W=0.99 | BP=15.79 |
| p-value | p=0.142 | p=0.902 | p=0.512 | p=0.066 |

***Suppl Table 8****: Multiple linear regression model tests for residual normality and model homoscedasticity.*
